# Bioelectricity in Developmental Patterning and Size Control: Evidence and Genetically Encoded Tools in the Zebrafish Model

**DOI:** 10.3390/cells12081148

**Published:** 2023-04-13

**Authors:** Martin R. Silic, GuangJun Zhang

**Affiliations:** 1 Department of Comparative Pathobiology, Purdue University, West Lafayette, IN 47907, USA; 2 Center for Cancer Research, Purdue University, West Lafayette, IN 47907, USA; 3 Purdue Institute for Inflammation, Immunology and Infectious Diseases (PI4D), Purdue University, West Lafayette, IN 47907, USA; 4 Purdue Institute for Integrative Neuroscience, Purdue University, 625 Harrison Street, West Lafayette, IN 47907, USA

**Keywords:** zebrafish, patterning, embryonic development, long fin, short fin, pigment, ion channels, bioelectricity, GEVI, optogenetics, chemogenetics

## Abstract

Developmental patterning is essential for regulating cellular events such as axial patterning, segmentation, tissue formation, and organ size determination during embryogenesis. Understanding the patterning mechanisms remains a central challenge and fundamental interest in developmental biology. Ion-channel-regulated bioelectric signals have emerged as a player of the patterning mechanism, which may interact with morphogens. Evidence from multiple model organisms reveals the roles of bioelectricity in embryonic development, regeneration, and cancers. The Zebrafish model is the second most used vertebrate model, next to the mouse model. The zebrafish model has great potential for elucidating the functions of bioelectricity due to many advantages such as external development, transparent early embryogenesis, and tractable genetics. Here, we review genetic evidence from zebrafish mutants with fin-size and pigment changes related to ion channels and bioelectricity. In addition, we review the cell membrane voltage reporting and chemogenetic tools that have already been used or have great potential to be implemented in zebrafish models. Finally, new perspectives and opportunities for bioelectricity research with zebrafish are discussed.

## 1. Introduction

Embryonic development is a self-autonomous and robust process in which a new body develops from a fertilized egg. This developmental process requires coordinated and complex cellular events such as proliferation, differentiation, and movement. The related patterning mechanisms are essential and instructive elements that eventually guide the body shape and organ sizes [1,2,3]. The morphogen gradient and transcription network are the mainstay theories and have been verified in many organ systems of various organisms [4,5,6]. Recent and past evidence revealed that ion-channel-related bioelectricity is a new component of the regulating mechanism for developmental patterning, regeneration, and cancers [7,8].

**Figure 1:**
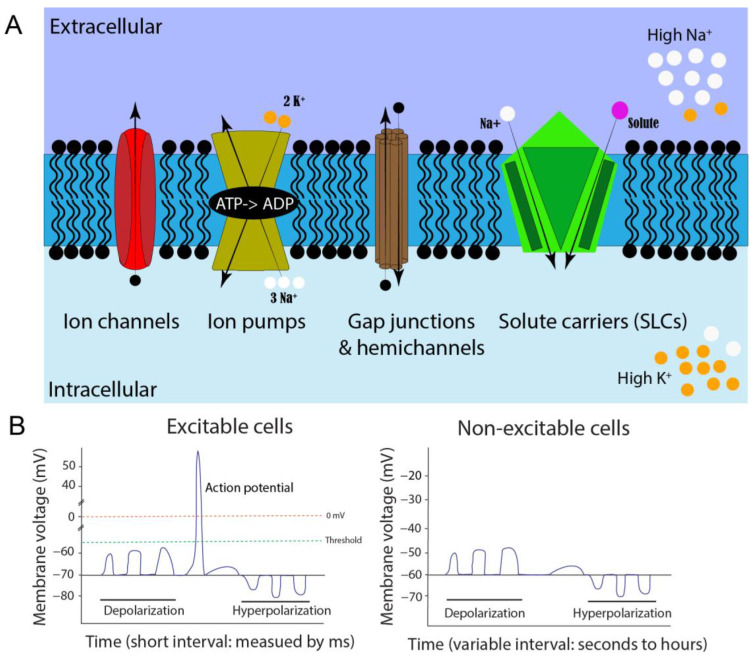
Cell membrane potential formation and comparison of neuromuscular excitable cells and non-excitable somatic cells. (**A**). Illustration of resting membrane potential, ion regulators, and ionic concentrations when the cell is in a non-excitable state. Different shapes represent various ion regulators on a cell membrane (blue region). The arrows indicate the movement of ions when the regulators are open. (**B**). Comparison of neuromuscular excitable and non-excitable somatic cells. Excitable cells usually exhibit action potentials, while the non-excitable somatic cells have membrane potential fluctuations, which vary in their amplitudes and frequencies.

Bioelectricity is defined as endogenous electrical signaling across cell membranes and is mediated by the dynamic distribution of charged molecules [7,8,9,10,11,12,13]. This is represented by a difference in the net charge of cations and anions inside versus outside a cell. Many components are involved in electrical potential formation [12,14]. In essence, the semipermeable lipid-based plasma membrane acts as an electrical insulator, but also as a capacitor that can accumulate charge, while specialized passages (ion channels, pumps, connexins/gap junctions, and solute carriers) regulate ion flow from one side to the other, altering the voltage of the cell (Figure 1A). All cell types form ionic gradients across their cell membranes because channels exist throughout all living organisms in all domains of life, including plants, fungi, and bacteria [14,15,16,17,18,19,20,21,22,23,24]. Thus, ion regulation and the resulting bioelectricity are considered essential properties of living cells across evolution, and their innate properties can be used for cellular communication [25,26]. Therefore, understanding additional aspects of bioelectricity in cells and organisms is fundamental for modern physiology and ontology.

Neuronal and muscular systems have been well investigated for their bioelectric activities. The field of neuromuscular bioelectricity has a relatively long and diverse history [27]. Luigi Galvani first demonstrated the relationship between electricity and animals in 1780 by electrically stimulating frog limbs to cause movement. However, it was almost another hundred years before the first measurements of action potentials, in 1865 by Julius Bernstein, using a differential rheotome [28]. The first intracellular electrical measurements of the resting membrane in the protozoon *Paramecium* were performed in 1934 [29]. Afterward, ion discoveries on neuronal bioelectricity were made by Hodgkin and Katz, using the giant squid axon as an experimental model [30]. Their intracellular recording studies paved the way for neurology and the fundamental understanding of action potentials [31]. One example is the combinational uses of neuronal axons’ action potential, voltage-gated Ca^2+^ ion channels, and synaptic neurotransmitters for neural signals [32]. However, the function of bioelectricity remains largely unknown outside of a neuromuscular context. Expanding on these concepts of neuronal bioelectricity and neurotransmitters, it is not inconceivable that other electrical signals could travel across the membranes of non-nerve cells and trigger various responses: to cause other ions to enter the cell (or be released from internal stores); to change transcriptional regulation of the machinery; to cause protein modifications, such as conformation or phosphorylation, to affect function; as well as to modify plasma membrane molecules such as receptors, kinases, and lipids [33,34,35,36]. Indeed, recent advances in bioelectricity research regarding embryonic development, regeneration, cancers, and potential mechanisms have been systematically reviewed [8,37,38]. In this review, we will focus on zebrafish mutants with patterning defects and genetic tools that are related to bioelectricity.

## 2. Cellular Contributors to Membrane Potential and Bioelectricity

### 2.1. Cell Membrane Potential and Concentration Gradients

Bioelectricity can be exhibited in several different forms in multicellular organisms: on cellular, tissue, and organ levels. For example, cell membrane potential or membrane voltage (Vm) is one of the integral cellular bioelectric properties (Figure 1A). Many essential cellular physiological processes rely on Vm. These include cross-membrane transport (e.g., nutrients, salts, water), cell volume control, secretion, the cell cycle, and migration [13,25]. Additionally, Vm allows for cognitive and motor function through neuronal signaling, resulting in organismal, tissue, or cellular sensory detection, and locomotive movement [25].

In typical neuronal signaling, the steady-state baseline voltage is called resting Vm, whereas the excited “signaling” state is called an action potential (AP). The resting cell membrane potential is the overall combination of ions for a cell, but the equilibrium potential for each ion is different in different cell types [25], resulting in a range of resting membrane potentials in each cell type (Table 1). Although this generally results in a range between −30 and −80 mV, Vm can even exceed a range of −5 mV to −150 mV, depending on cell type [9]. These resting Vm values can fluctuate in a small or large deviation. Large and rapid depolarization changes from negative to more positive membrane potential are referred to as APs, which are barely reported outside of neuronal and muscular tissues. These APs are triggered by ion channels that respond to changes in voltage that reach a certain threshold. More specifically, depolarizations may merge along a neuron axon or dendrite, eventually pass the Vm threshold for voltage-gated ion channels, and form an AP [39]. These AP waves can propagate from multiple locations, and if two meet from opposing directions, they will annihilate each other [40]. This quick (millisecond) and extreme (≥100 mV difference) swing in voltage, caused by altering intracellular ion concentrations, is unique to excitatory cells. However, increasing evidence shows smaller and longer-duration types of electrical signaling events in other, non-excitable cell types, such as melanocytes, can have significant effects [41]. Changes in Vm of non-excitable somatic cells could come from a variety of factors and would not be classified as traditional AP signals (Figure 1B). Smaller and less extreme increases or decreases in Vm can occur within embryonic neural and non-neural tissues over various periods, such as milliseconds, seconds, minutes, hours, or even days. Such subtle bioelectric signals may be essential in cell differentiation and embryonic patterning during development [7,8,9,37].

The electromagnetic force of the differential distribution of ions across the cell membrane generates the electric potential. Thus, the concentration gradient of each ion molecule jointly contributes to Vm value [63]. For example, there is a high level of potassium (K^+^) and low levels of sodium (Na^+^) within cells at resting Vm. High levels of intracellular K^+^ and extracellular Na^+^ ions are mainly established by the sodium/potassium ATPase pump (Figure 1A). One ATPase pump binds three intracellular Na^+^ ions, utilizes ATP to change conformation via phosphorylation, and releases the three Na^+^ ions into the extracellular space. Next, two extracellular K^+^ ions will bind to this outward-facing conformation, causing dephosphorylation and reversal of conformation that allows potassium ions into the cell against its concentration gradient [7,64]. This form of active transportation and the resulting electrochemical gradient is responsible for high intracellular potassium.

The electrical potential difference that counteracts or balances the concentration gradient for a given ion is called equilibrium potential. If only one permeant ion species exists in a cell, its resting membrane potential will equal the equilibrium potential for that ion. Potassium and sodium ions are the two main contributors to membrane potential, but Cl^−^ and Ca^2+^ ions can also affect Vm, in addition to other charged molecules, such as protons (H^+^) and organic anions, depending on cell types. Generally, potassium equilibrium potential is close to resting cell membrane potential in many cell types, including glia and neurons. Thus, maintenance of high intracellular potassium is critical for establishing resting Vm [32,63]. This difference in concentration is hard to maintain, and potassium ions can exit the cell through various leak channels, such as K2P potassium channels on the plasma membrane [65]. Removing positively charged K^+^ ions from the cell will result in a more negative electrical charge, forcing more positive ions to be pulled back into the cell against the chemical gradient. This constant cycling of potassium being pumped into cells and leaking out helps to establish the electric potential of resting Vm. Eventually, these electric and gradient forces will reach equilibrium. This balance can be mathematically described in the Nernst equation [32,63].

### 2.2. Membrane Potential Contributors: Ion Channels, Gap Junctions, and Solute Carriers

Ion channels are a group of transmembrane proteins that significantly contribute to overall cellular bioelectricity. Channels are essentially small pores in the cell membrane that alter permeability for specific ions based on selectivity (molecular charge and size) and gating (what is required to open the channel) [66]. Channel conductivity is aligned with the ion concentration gradient, so energy is not required for a high rate of ion-selective transport. However, the channels will only allow ions to flow down their concentration gradient (moving from high to low concentration areas). The composition of these channels on the cell membrane has been compared to an electronic component called a field-effect transistor [67]. In the human genome, more than 400 family members of ion channels are currently characterized, accounting for around 1.5% of the genome [68]. A comprehensive list of human ion channel details can be found on the HUGO Gene Nomenclature Committee website and the IUPHAR/BPS Guide to Pharmacology [66,69].

Based on ion selectivity, ion channels can be classified as sodium (Na^+^), calcium (Ca^2+^), potassium (K^+^), chloride (Cl^−^), or proton (H^+^) channels, as well as non-charged molecules such as aquaporins [66,70]. The most direct Vm-contributing ion channels are K^+^ and Na^+^, while the others play a minor role or secondary messenger role, such as that of Ca^2+^. Each ion channel type can then be further categorized by gating mechanism. One group, voltage-gated channels, will open or close when their voltage-sensitive domains detect a specific change in membrane potential, usually a significant depolarization from action potentials in neurons. Another type, ligand-gated ion channels, relies on their receptor binding a particular ligand to cause or prevent ionic flow. A third category, leak channels, continually allows a small amount of sodium or potassium to leave the cell, regardless of Vm state [65,71,72]. This type of channel can profoundly impact Vm because it can heavily affect the ion gradient at different stages of excitatory conditions. There are additional mechanisms to regulate or gate leak channels, such as temperature, mechanical force, and light [65,71,72]. Another interesting group of channels is that of inwardly rectifying potassium channels (Kir) [73]. These channels allow K+ ions to move more easily into, rather than out of, a cell when the cell membrane is depolarized. This is because the intracellular concentration of potassium is so high at rest, and this type of ion movement occurs against the concentration gradient. Even when these are functioning, it is difficult for K+ ions to enter the cell, and they might leak out. Due to this unique characteristic, the Kir channels will impact concentration gradients, resting membrane potential, and cell excitability [73]. Furthermore, different channels can show distinct levels of rectification (e.g., high or low). The lipid species, such as PIP2 (phosphatidylinositol 4,5-bisphosphate), can further regulate Kir channels, as can Mg^2+^, polyamines, phosphorylation, or protein–protein interactions [73].

Gap junctions are membrane proteins that physically connect adjacent cells to allow ions, small molecules, and electrical impulses to pass directly by a regulated gate between cells. Like ion channels, their conductance is passive and down an electrochemical gradient. Thus, they do not rely on ATP-like ion pumps. Gap junctions are formed by connecting proteins called connexins and pannexins in vertebrates and innexins in invertebrates (depending on the number of Cys residues in their extracellular loop and glycosylation) [74]. These connexins have unique protein structures, properties for permeability, and gating. Each gap junction comprises six connexin subunits on one cell that oligomerize with another six connexins on an adjacent cell. The connection of the same connexin isoform is called homogenous/homomeric, but these properties can change and become more complex by forming heterogeneous/heteromeric gap junctions [75]. When these connexins are not coupled to form a gap junction, they are known as hemichannels [76]. These hemichannels may serve as an ionic and molecular interchange routes between the cytoplasm and the extracellular environment [77]. Gap junctions and hemichannels play significant roles in cell-to-cell communication by exchanging ions, small molecules, subcellular vesicles, electric impulses, and organelles, due to their relatively larger pores [78]. Thus, they are natural modulators of cellular bioelectricity. Electrical synapses between neurons can be considered a specialized gap junction. In addition, gap junctions have also been found to be needed for direct cell communication in tunneling nanotubules (TNTs) [79,80]. Gap junctions are crucial for many physiological processes, including synchronized depolarization of cardiac muscle and embryonic development [81,82]. One ubiquitous gap junction, connexin 43 (CX43), has been implicated in multiple organisms and diseases and it contributes to electrical signaling [83]. Connexin mutations and misregulations have been shown to cause many diseases, such as neurodegenerative diseases and congenital morphological defects in mice and humans [84,85].

Another group of Vm ion regulators is solute carrier proteins (SLCs). These proteins utilize secondary active transport, where thermodynamically favorable reactions (i.e., ions moving down their concentration gradient) are paired with one or more other molecules to be transported in an unfavorable reaction [86]. The free energy provided by the movement in the favorable direction makes movement in the less favorable direction possible and allows transport without directly consuming cellular energy. These reactions utilizing the electrochemical gradient can occur with both substrates moving in the same direction, known as symporters, or substrates moving in opposite directions, known as antiporters. Thus far, over 450 transporter proteins are found in the plasma membrane of cells and subcellular organelles [86,87,88]. These SLCs have an extensive range of substrate specificity, including ions, organic ions, sugars, vitamins, amino acids, nucleotides, oligopeptides, drugs, and metals. In addition, some SLCs can transport multiple different biomolecules, others can only transport a single biomolecule, and up to 30% are “orphan” proteins, whose substrates remain unknown. Thus, these SLCs have been involved in many physiological regulations, such as selective barriers, neurotransmitters, nutrition, and metabolic regulation [86,88]. More than 190 diseases have been linked to SLCs, such as thyroid, hearing, neurological, metabolic, and congenital defects [86,88]. Due to the nature of their substrates, the SLCs could be an essential contributor to cellular bioelectricity.

## 3. Bioelectricity Evidence from Zebrafish Genetics

### 3.1. Zebrafish as a Superior Model for Bioelectric Research

The zebrafish has become one of the leading model organisms used in research since its debut in the 1970s, due to its unique advantages [89,90,91]. First, zebrafish share vertebrate biology with humans. Zebrafish possess 70% orthologous genes to humans [92]. Second, it is a relatively affordable model, compared to murine models. Third, small body size and external development make zebrafish embryos an ideal in vivo system. Fourth, tractable genetics has been developed in zebrafish, including large-scale forward genetic mutagenesis, CRISPR-based reverse genetics, and Tol2 transposon-based transgenesis [93,94,95]. Furthermore, a significant source of mutation lines is available through the repository ZFIN, and the greater zebrafish research community is highly collaborative [96,97]. All these advantages make zebrafish popular for studying developmental biology, neuroscience, physiology, toxicology, drug screens, and many human diseases such as cancers [89,90,91,98,99]. Zebrafish are also particularly suited to bioelectric research. The combination of excellent and well-established genetic tools with transparent external embryonic development can allow for manageable mutant generation and cutting-edge microscopy to explore previously unattainable information. These attributes can also be useful in bioelectric research. Below, we highlight bioelectric-related zebrafish studies that demonstrate the importance of this model as an optimal way to characterize and uncover the as-yet-undetermined bioelectric characteristics and mechanistic properties.

### 3.2. Zebrafish Mutants with Adult Fin-Size Change

Zebrafish adults have two sets of fins: paired fins (pectoral and pelvic) and unpaired median fins (dorsal, anal, and caudal), aligning their anterior to posterior body margins [100,101,102]. Each fin comprises endoskeletons and external dermal bones: the fin rays, or lepidotrichs. The adult fin size and its proportion to the body are generally unvarying. Zebrafish paired fin development was reported to share similar mechanisms with tetrapod limbs, as corresponding signaling centers such as ZPA (zone of polarization) and AER (apical ectodermal ridge) were characterized in zebrafish [100,103,104]. Although direct evidence of bioelectricity in zebrafish fin development is still lacking, indirect evidence came from several zebrafish fin mutants from large-scale forward genetic screenings. These mutants display either elongated or short fins, and underlying mutated genes are involved in normal ion regulation via channel, solute carrier, or connexin (Table 2), indicating that bioelectricity, not a specific ion regulator, is the key to zebrafish fin patterning and size regulation.

The first reported zebrafish mutant with elongated fin size is *longfin*(*lof^t2^*), which is a dominant mutant that occurred in nature and is present in the widely used Tüpfel fish line. The causal mutant gene of the *lof* has remained unknown for decades until recently. Two independent reports pinpointed Kcnh2a, a voltage-gated potassium channel [105,106]. There is a 0.9 Mb chromosomal reversion upstream of the *kcnh2a* gene on chromosome 2 [106]. This inversion disrupts gene regulation and causes a change of the cis-ectopic expression of *kcnh2a* in zebrafish fins. Similar to the *lof^t2^*, another longfin (*alf^dty86d^*), an ENU-induced mutant, possesses elongated fins in adults in a dominant way [107]. This *alf*^dty86d^ mutant was reported to be caused by gain-of-function mutations in *kcnk5b*, a potassium leak channel gene [107]. The authors also reported larva fish overgrowth and cellular voltage change, indicating the Kcnk5b-mediated bioelectricity of fin anlagen could be the underlying mechanism through local overgrowth [107].

The *schleier* is another zebrafish mutant with elongated fins. This mutation is caused by the inactivation of a potassium–chloride cotransporter, *slc12a7a/kcc4a* [108]. This mutant is also genetically dominant, and homozygous adults exhibit broken stripes and pigmentation alternations. A CRISPR mutation experiment revealed that the function levels of Kcc4a correspond to the fin and barbel lengths. In addition, *kcnk5b* knockout in the *schleier* fish embryos can reduce the adult fin lengths, suggesting that Slc12a7a might function together with Kcnk5, and both might be required for bioelectric regulation in wildtype fish. Interestingly, the same research group also identified *slc43a2/lat4a*, an L-leucine amino acid transporter that can modify the *kcnh2a* mutation effect in *lof^t2^* mutant fish, resulting in a flying-fish-like phenotype [106]. This *lat4a* mutant, *lat4a^nr21^*, is also dominant and exhibits a short-finned phenotype in heterozygotes. The interactions between Lat4a and Kcnh2a in the flying-fish-like zebrafish suggest they are also involved in bioelectric regulation. Along with this short-finned phenotype, two additional mutants were reported. They are the shortfin (*sof*) mutants (4 alleles: *sof^b123^* (spontaneous)*, sof^j7e1^, sof^j7e2^, sof^j7e3^* (ENU-induced)) caused by a hypomorphic mutation in the gap junction, Cx43 [109], as well as a fish mutant, *mau*, caused by a dominant missense mutation in *aqp3a* (*aquaporin 3a*) [110]. Like the *lat4a^nr21^* mutation, the *cx43* mutation in *sof* also reverted the *lof^t2^* long-finned phenotype [111], suggesting that Cx43 is another bioelectric regulator for zebrafish fin size.

Our laboratory recently characterized a dominant long-finned mutant, Dhi2059, which was generated via a large-scale insertional mutagenesis [112]. The *kcnj13* gene’s exon 5 was disrupted by a retroviral insertion. Although this exon encodes 5′ UTR (untranslated region), not protein, viral DNA insertion leads to a transient and ectopic expression of *kcnj13* in the somites between 15S (15-somite stage) and 48 dpf (days post fertilization) in Dhi2059 fish embryos. Transgenic fish Tg (−5.4k *pax3a*: *kcnj13*-IRES-EGFP), in which the *kcnj13* gene is under the control of the −5.4k *pax3a* promoter, can phenocopy the long-fin phenotype. Thus, *kcnj13* misregulation resulted in elongated fins in the adult zebrafish, mainly by increasing the length of fin rays [112]. Different from the previously mentioned long-finned mutants (*lof, alf, schleier*), our results suggest that the adult fin size can be determined at the somite stage in early fish embryos. This indicates that bioelectricity is set up early and could serve as a memory for patterning and size regulation in later ontology (see detailed discussion in the prospective section). In addition, we showed that transient expression of multiple potassium channels (*kcnj1b, kcnj10a, kcnk9*, human *KCNJ13*) in zebrafish early embryos (by microinjection) could also cause chimeric long fins in injected adult fish. This result suggests it is not a specific potassium channel, but that bioelectricity is the key to the elongated fin phenotype.

Multiple key points can be obtained by comparing these zebrafish mutants. First, all the mutant genes are involved in ion regulation, which is intrinsically linked to bioelectricity. These ion regulators have their own ion type selecting properties and conductance. It becomes challenging to explain the fin phenotype with a specific channel or ion. Instead, it is more reasonable that electric signaling is the underlying mechanism. Different ion regulators with other properties can be used to construct and modify the bioelectric state of cell groups and tissues. Second, all of these mutations are genetically dominant; most are gain-of-function, ectopically expressed, or neomorphic. Lastly, the specificity of the zebrafish fin-size phenotype may be caused by the spatiotemporal distribution of these ion regulators during embryonic development, as exampled by the Dhi2059 mutant. Taken together, the zebrafish’s adult fin size could be regulated at multiple stages. Although most studies reported altered gene expression in fin anlagen or local fins, our experimental data suggested that somites, the embryonic origin of fin ray progenitor cells, can play a critical patterning role.

Consistent with zebrafish mutants, it is also worth noting that different potassium channels were recently identified in other teleosts through genome association studies. The inwardly rectifying channel gene *kcnj15* was mapped to long-finned betta fish [113]. Additionally, the ether-à-go-go (EAG) potassium channel gene, *kcnh8*, was found to be highly expressed in the male caudal fins in Xiphophorus [114]. Like zebrafish, *kcnk5bS* was identified as a candidate for long-tailed goldfish [115]. Together with zebrafish mutants, these data suggest that ion-channel-mediated bioelectricity plays an essential role in fin patterning.

**Table 2:** Zebrafish, published fin-size mutants.

Mutant	Fin	Gene	Mutation Nature	Fin Ray Segment Length	Fin Ray Numbers	Dominant or Recessive	Somite or Local Fin	References
*lof*	Long	*kcnh2a*	Ectopic by cis-regulatory change	Normal	Increased	Dominant	Local	[105,106]
*alf*	Long	*kcnk5b*	GOF	Increased	Decreased	Dominant	Local	[107]
*schleier*	Long	*slc12a7a/kcc4a*	LOF or dominant negative? Dose dependent	Normal	Increased	Dominant	Local	[108]
*Dhi2059*	Long	*kcnj13*	Ectopic by cis-regulatory change, Dose dependent	Increased	Decreased	Dominant	Somite	[112]
*sof*	Short	*cx43*	Hypomorphic	Decreased	Decreased	Dominant	Local	[109]
*mau*	Short	*aqp3a*	Neomorphic, Dose dependent	Normal	Decreased	Dominant	Local	[110]
*nr21*	Short	*slc43a2/lat4a*	GOF	Decreased	Not reported	Dominant	Local	[111]

Note: GOF: gain-of-function; LOF: loss-of-function.

### 3.3. Zebrafish Mutants with Adult Pigmentation Pattern Alterations

Zebrafish adults exhibit distinct stereotypical stripe patterns along their bodies, with alternating rows of melanophores (dark pigments) and xanthophores (red-orange pigments) mixed with iridophores (iridescent pigments) [116,117,118]. Local and long-range interactions and communication among these different pigment cells during embryonic and larval stages are essential to forming the stripe patterns [79,115,116]. Among many mutant zebrafish lines with altered pigmentation patterns, several are mutations of ion regulators, suggesting that ion-channel-mediated bioelectric signals play important roles in pigmentation patterning. Two of the fish mutants, *albino* and *golden*, resulted from the loss of function of solute carrier genes, *slc45a2* and *slc24a5*, respectively [119,120,121]. The phenotypic results of the two mutants are a complete loss of melanophores and light stripes (melanophores with small and fewer melanin granules), respectively. The two genes are expressed in zebrafish melanophores, and light pigmentation was thought to be mainly caused by reduced melanogenesis due to ion and proton alteration in the melanophores [120,121]. The *transparent* (*tra*) fish possess fewer iridophores, melanophores, and dark spots, instead of stripes, in adults. This *tra* is a loss-of-function mutation of the *mpv17* gene, which encodes a non-selective channel that modulates mitochondria membrane potential [122,123]. Although the loss of Mpv17 was found to cause a reduction in the number of mitochondria and reduced pyrimidine synthesis [123], the bioelectricity of iridophores might also contribute to patterning defects.

In addition to chromophore defects, zebrafish stripe patterns were found to be altered in additional mutants. The *leopard* (*leo^t1^*) mutation, also known as *tup*, is a spontaneous recessive mutation causing spots in the adult *Tüpfel* fish line. This mutation is caused by the *cx41.8* (*connexin 41.8*) gene, which encodes Gja5b in zebrafish [124]. Similarly, *luchs* (*luc^tXA9^*) is a mutation of the *cx39.4* (*connexin 39.4*) gene, which encodes Gja4 [124]. Both *cx41.8* and *cx39.4* are required for melanophore and xanthophore development. Both mutants show aggregated dark spot patterns instead of stripes. Interestingly, it was shown that these two connexins could form heteromeric, in addition to homomeric, gap junctions, which are essential for melanophore and xanthophore cellular communication [124,125]. Recently, another mutant zebrafish, *schleier*, was reported to be caused by hypomorphic function of another solute carrier, *slc12a7a/kcc4a* [108]. The homozygous mutant fish show broken stripes in the ventral body flank and anal and caudal fins. Gap junctions usually conduct small molecules and ions between neighboring cells. Thus, they can modulate molecular and electrical coupling among the adjacent cells [81,82], and over longer distances [79,80]. Additionally, the *obelix(obe)/jaguar(jag)* mutants, which are caused by a *kcnj13* loss of function, have fewer stripes compared to wildtype fish [126]. Kcnj13 is an inwardly rectifying potassium channel that regulates cell excitability and membrane potential. Based on the less severe pigmentation phenotype of the *kcnj13* null mutants (*kcnj13^pu107^, kcnj13^pu109^*) our lab generated, the original alleles (*jag^b230^, obetc^271d^, and obe^td15^*) are most likely dominant negative [112,126]. More recently, *kcnj13* expression was found to underlie the pattern diversification among *Danio* species via the *kcnj13* regulatory changes [127]. This potassium channel gene is expressed in melanophores during development, suggesting that it may regulate melanophore bioelectric properties. Indeed, cellular electrical communication was partially disrupted in this mutant. The dissociated melanophores of *jag* are more depolarized when measured with a voltage-sensitive dye, DiBAC4(3), than the melanophores from wildtype fish. Wildtype melanophores are transiently depolarized when contacted by the dendrites of a xanthophore, and then moved away from the xanthophore. In contrast, *jag^b230^* melanophores lost contact-dependent depolarizations and repulsive migration behavior [128].

Three additional zebrafish mutants could also be related to bioelectric regulation, though the related genes are not direct ion regulators. Spermidine is an endogenous polyamine that can regulate ion channels and connexins [129,130]. The *idefix* (*ide^t26743^*) fish is a loss-of-function mutant of the *srm* (*spermidine synthase*) gene [131]. Homozygous *ide^t26743^* mutants have fewer narrowed and often interrupted dark stripes in the trunk and fewer strips in the fins. This *ide* mutation can further reduce melanophores when crossed with *leo^t1^*, *luc^tXA9^*, and *obe^271d^* mutants, suggesting that spermidine may modulate connexin and potassium channel functions. Moreover, ectopic expression of spermidine/spermine N1-acetyltransferase (Ssat), a polyamine metabolic enzyme in melanophore, caused broken stripes and a loss of melanophores in the *leo^t1/t1^* background, also supporting this idea [132]. Another zebrafish mutant, *schachbrett* (*sbr^tnh009b^*), is caused by a loss of function mutation of tight junction protein 1a (Tjp1a), which is expressed in iridophore [133]. Like *ide^t1^*, the *sbr^tnh009b^* mutant exhibits more substantial pigment patterning defects in *luc^t32241^* and *leo^t1^* background, indicating Tjp1a may interact with connexins. Thus, Tjp1a may indirectly affect the bioelectricity of chromatophores. The third zebrafish mutant, *mau,* also possesses spotted pigments. The underlying gene of the *mau* mutation is *aqp3a,* which is mainly expressed in skin and muscle, but not in chromatophores [110]. Transplantation of *aqp3a^tVE1/+^* blastomere cells into wildtype and Aqp3a^R220Q^ in a transgenic experiment revealed that Aqp3a might indirectly influence chromatophores for pigment patterning. Aqp3a is a transporter of non-polar solutes such as glycerol, peroxide, and urea, excluding ions [134]. Thus, Aqp3a can modulate the ion concentrations related to cellular bioelectricity.

## 4. Genetically Encoded Tools That Can Be Used for Studying Developmental Bioelectricity

Functional studies of the bioelectric mechanisms of embryonic developmental processes require suitable tools with which to measure endogenous bioelectricity in a real-time and non-invasive manner and manipulate cell and tissue bioelectricity without compromising whole embryo tissue integrity. With recent significant advances in neuroscience, various genetically encoded tools were developed to meet these purposes. Genetically encoded indicators, or biosensors, can allow us to measure cellular membrane potential, ion concentration, and even metabolites via fluorescence [135,136]. Additionally, chemogenetic and optogenetic tools allow us to manipulate cellular bioelectricity, such as membrane potential, in a precise manner [137,138,139,140,141,142,143]. Although initially developed for studying neurons, these tools can also be utilized in other research contexts, such as for studying bioelectricity in embryonic development.

### 4.1. Measuring Cellular Bioelectricity: Genetically Encoded Voltage Indicators

Cellular electric neural activity can be accurately measured using traditional electrode measurements, such as the patch clamp method. This method is highly accurate, but is generally limited to single-cell recordings and invasive to cells [144,145,146]. Calcium has also been widely used to reflect neuronal electric activities. Genetically encoded calcium indicators, GECIs, have been widely implemented into the zebrafish model for neural studies [147,148,149], cell migration [150,151], embryogenesis [152,153], insulin secretion from pancreatic beta cells [154], and many other biological processes [155,156,157,158]. Although calcium signals mimic electric signals, they are still different due to their additional function as a secondary messenger in various cell types. Indeed, a difference was reported in calcium and voltage signals [159,160]. Genetically encoded voltage indicators (GEVIs) were invented to directly measure neurons’ electrical activity, complementary to patch clamp electrophysiology. These non-invasive, endogenous fluorescent biosensors can function over multiple cells and tissues to provide a collective understanding of real-time bioelectric activities versus single cells. These are also more advantageous over previously developed electrochemical dyes due to their increased speed, genetic specificity, higher sensitivity, and lack of toxic effects [161]. The fastest GEVIs have reported rates up to 1 ms [135]. Another advantage of GEVIs is the ability to provide results over extended periods. While these sensors offer several advantages, they do have some drawbacks, such as genetic modification and the high-end fluorescent microscopes required, variable dynamic ranges, and signal-to-noise ratios [135]. However, with the advance of technical developments, these shortcomings are being overcome, and GEVI applications are expanding beyond neuroscience into many fields, such as developmental biology.

**Figure 2:**
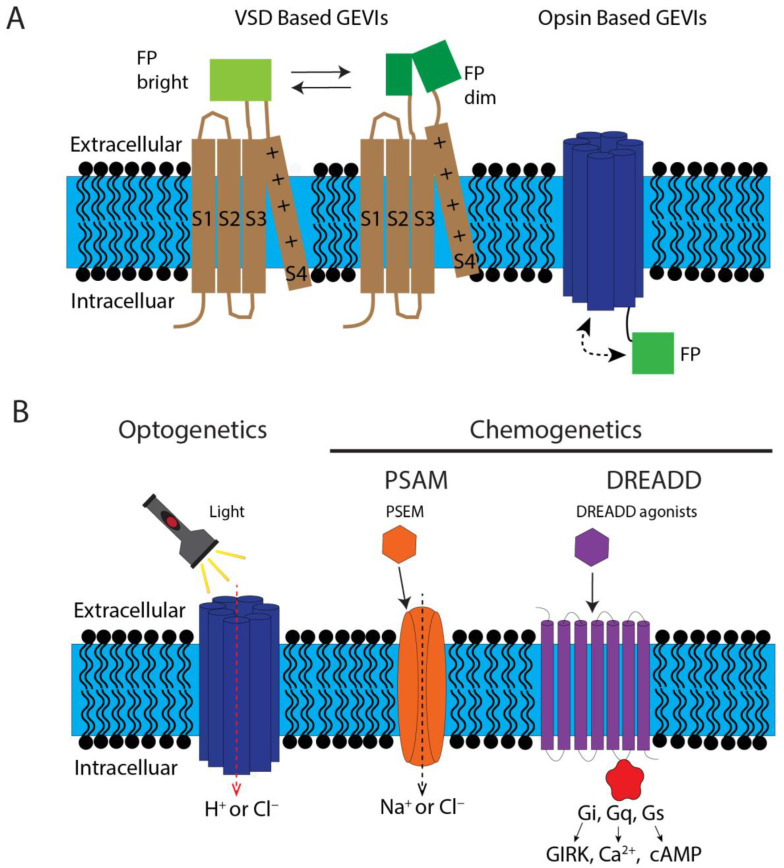
Illustrations of design principles of common GEVIs, optogenetic and chemogenetic tools. (**A**). Schematic structures of VSD (voltage sensitive domain) based and opsin-based GEVIs. The voltage-sensitive domain is labeled brown in the cell membrane. A fluorescent protein (FP) is inserted into the S3–S4 loop. When Vm is altered, the fluorescence intensity will change accordingly. The light-sensitive opsin (dark blue) can sense the Vm of the membrane and act as a chromophore. Thus, it can be used to measure the Vm with or without a connected FP, which can enhance the overall signal. (**B**). Principles of Optogenetics and chemogenetics. The optogenetic tools are based on light-sensitive channel rhodopsins that conduct protons or chloride. The PSAMs are mutated ligand-gated ion channels for sodium or chloride. They are controlled by artificial PSEM (pharmacologically selective effector molecules) ligands. In contrast, the DREADDs are mutated ligand-gated GPCRs (G-protein-coupled receptors). Depending on the type of G protein, they can increase or decrease Vm via GIRK channels, calcium signaling, and cAMPs. The arrows indicate the movement of ions when the regulators are open.

Thus far, numerous advancements and variations of GEVIs have been developed (Table 3). These GEVIs usually fall into one of three categories (Figure 2A) [135,162,163]: (1). GEVIs based on a voltage-sensitive domain (VSD) within the cell membrane, usually from the tunicate (*Ciona intestinalis*) voltage-sensitive phosphatase, PTPE [164,165]. The VSD can be linked with either a single fluorescent protein (FP), dual FPs for FRET (Forester resonance energy transfer) signaling, or even bioluminescence. (2). Opsin-based GEVIs, with and without additionally combined FPs to improve brightness. (3). There is a group of hybrid GEVIs that combine these different components with the addition of brighter and more photostable synthetic dyes [135,162,163]. Each GEVI has its unique properties and application niche. Many of these GEVIs have also been examined and utilized in zebrafish research (Table 3).

One commonly used GEVI is ASAP (accelerated sensor of action potentials) based on VSD design. A circular permutated GFP is inserted int the middle of S3–S4 loop of the VSD. Thus, when the VSD protein confirmation is altered by Vm, the intensity of GPF fluorescence will change correspondingly (Figure 2A). When the cell membrane is hyperpolarized, the ASAP1 fluorescence signal is brighter. This ASAP1 was successful, and neuron bioelectricity has been well-documented in many model organisms, including zebrafish. A few updated versions have also been developed to improve its speed, signal-to-noise ratio, and sensitivity [166,167,168,169]. Our lab has generated a ubiquitous transgenic reporter zebrafish line Tg(*ubi*: *ASAP1*) [170,171]. With this ASAP1 transgenic fish line, real-time endogenous cellular bioelectric activities can be visualized in fish embryos, larvae, adults, and even tumor tissues [170]. Our results are consistent with an independent report on Tg(*UAS: ASAP1*), a binary transgenic fish line for tracking larval fish neuronal circuitry within cerebellum, optic tectum and spinal cord [172,173]. In addition, we made new observations in early fish embryos. We found that a transient local membrane depolarization occurs before cleavage furrow formation during zebrafish embryo cleavage stages (1–64 cells). This phenomenon is consistent with calcium signaling measured by GCaMP6s [153]. These Vm changes are not static, but dynamic during the cell division period. Moreover, these Vm dynamic changes are not perfectly synchronized among early cells. These results suggest a biological function of Vm in cell division. Membrane potential changes have been shown to influence the organization of phospholipids. These are known as critical components of the cleavage furrow and cytokinesis [36,174,175]. Once zebrafish embryos enter the blastula stage, the bioelectric signals become whole-cell transient hyperpolarizations, mainly found in the rapidly dividing superficial layers of the blastula (EVL) and yolk syncytial layer (YSL). During gastrulation, Vm transients continued in the EVL and YSL, and started to occur in the deeper cells. Moreover, we noticed differential Vm among different embryonic tissues and somite-specific hyperpolarization events during the zebrafish embryonic segmentation period. These results demonstrated that the ASAP family and potentially other GEVIs could be readily used for measuring embryonic bioelectricity. We expect more bioelectric biology to be revealed by organ- and/or tissue-specific zebrafish transgenic fish lines. For example, different cell types in the zebrafish fins, pigment cells, and the other cell types in the skin can be characterized for their physiological bioelectric properties (for details, see the prospective section).

It is worth noting that GEVIs are not limited to zebrafish. They have also been applied to other organisms. For example, multiple studies have shown the utility of genetically encoded indicators in fruit flies, but have, thus far, only been focused on neuronal-related studies [167,169,176,177,178]. In mice, fewer research studies have utilized GEVIs in vivo, and most of these studies focused on neurological research [178,179,180,181,182]. In addition, *Xenopus* oocytes were used to characterize Arclight, but did not address any developmental biology [183]. Nevertheless, their results showed that these sensors could be employed to characterize bioelectricity in *Xenopus* if needed.

**Table 3:** List of published genetically encoded voltage reporters.

GEVIs	Fluorescence Indication	Fluorophore	Tested in Zebrafish	References
VSD-based				
ASAP1–3	Hyperpolarize—brighter	GFP	Whole fish embryos and larva. Adult malignant nerve sheath tumors, larval fish cerebellum, spinal cord.	[166,167,168,169,170,171,172,173]
ASAP4	Depolarize—brighter	GFP		[184,185]
Marina	Depolarize—brighter	GFP		[186]
FlicR1	Depolarize—brighter	RFP		[187]
Arclight	Hyperpolarize—brighter	GFP		[188,189]
Bongwoori	Hyperpolarize—brighter	GFP	Larval olfactory bulb	[190,191]
Aahn	Hyperpolarize—brighter (external)	GFP		[192]
VSFP x	Depolarize—FRET increase	Multiple	Larval zebrafish heart	[193,194,195,196,197,198,199,200]
Mermaid	Depolarize—FRET increase	Multiple		[201]
Nabi	Depolarize—FRET increase	UGK/mKO		[202]
JEDI-2P	Hyperpolarize—brighter	GFP		[178]
Opsin-based				
Arch	Depolarize—brighter	GFP		[203]
QuasAr x	Hyperpolarize—brighter	Multiple	Larval zebrafish heart	[204,205,206,207,208]
Archon1	Depolarize—brighter	GFP/RFP	Brain and spinal V3 interneurons	[209,210]
Ace x	Hyperpolarize—brighter	Green/RFP		[211,212]
Ace-mNeon2	Hyperpolarize—brighter	GFP		[182]
VARNAM	Hyperpolarize—brighter	RFP		[213]
VARNAM2	Hyperpolarize—brighter	RFP		[182]
pAce	Depolarize—brighter	GFP		[182]
pAceR	Depolarize—brighter	RFP		[182]
Dye- or bioluminescence-based				
Voltron	Hyperpolarize—brighter	Multiple dyes	Larval brain	[214]
Voltron2	Hyperpolarize—brighter	Multiple dyes	Larval olfactory sensory neurons	[215]
Positron	Depolarize—brighter	Multiple dyes	Larval zebrafish brain	[216]
hVOS	Depolarize—brighter	Green dye		[217]
Voltage spy	Depolarize—brighter	Green dye		[218]
LOTUS	Depolarize—FRET increase	Blue/green bioluminescence		[219]
AMBER	Depolarize—voltage-gated luciferase increase	Blue/green bioluminescence		[220]

### 4.2. Manipulate Cellular Bioelectricity: Optogenetic and Chemogenetic Tools

Another requirement to elucidate the bioelectric signaling mystery is the direct and specific perturbation of the normal electrical state of cells and tissues. As this was a major task for neuroscience, optogenetic and chemogenetic tools were already developed as experimental approaches (Figure 2B and Table 4). These tools have, thus far, demonstrated the capability to alter cell-specific electrical states of neurons to hyperpolarization and depolarization, allowing a precise level of control in various organisms [137,138,139,221,222,223].

*Optogenetics:* Optogenetics modulates bioelectricity through microbial (type I) opsins, which are light-sensitive ion pumps and ion channels found in prokaryotic and eukaryotic microbial organisms [224]. These type I opsins can conduct cations or anions under the control of different wavelengths of light [225,226,227]. When exposed to specific wavelengths of light on expressing cells, channels open to allow specific ions, such as H^+^, Na^+^, Ca^2+^, or Cl^−^, into cells, resulting in increased or decreased Vm. The use of optogenetics in zebrafish has been primarily targeted in neuroscience studies. These have been used to modify swimming behavior [228] and locomotion behavior [229], perturb hair cell sensory receptors [230], axon guidance control [231], and alter olfactory responses [232]. Optogenetic tools have also been used for other zebrafish research fields, such as heart physiology [233,234], and melanophore patterning [235]. In the zebrafish melanocyte study, ChR2 was expressed in the melanophores of zebrafish that were then placed in tanks exposed to blue light to stimulate depolarization [235]. As a result, these transgenic fish began to lose the boundaries of their standard stripe patterns [235]. Interestingly, this was partially reversed after allowing the depolarized cells to return to their average membrane potential, suggesting that endogenous bioelectric signals are essential for maintaining pigment homeostasis. This study also provided direct evidence of bioelectric signals in zebrafish pigment patterning. Recently, a set of optogenetic transgenic zebrafish lines have been created under the control of the UAS promoter [141]. This will accelerate bioelectric research in zebrafish. Moreover, with the great success of light-sensitive rhodopsin, new optogenetic tools have been invented with which to control gene expression, protein localization, and activity, using a new set of light-sensitive proteins such as phytochromes, blue light using flavin (BLUF) domain photoactive proteins, cryptochromes (e.g., CRY2-CIB1), and light oxygen voltage (LOV) domain proteins. Their applications to developmental biology and zebrafish have already been reviewed [236,237]. These new optogenetic tools are also utilized for investigating zebrafish bioelectricity.

*Chemogenetics:* Chemogenetics is a genetic approach to perturb cellular electrical activity using synthetic molecules through either mutated G-protein-coupled receptors (GPCRs) or ligand-gated ion channels that no longer function normally, but only in the presence of inert molecules [222,223].

DREADDs (Designer receptors exclusively activated by designer drugs) are one of the mainstream chemogenetic tools that are commonly used in neuroscience, including in the study of behavior, circuits, and diseases [222,238,239,240]. DREADDs are designed based on mutated muscarinic and opioid receptors. Four types (hM3DGq, hM4DGi, hM3DGs, and KORD) alter cellular Vm through downstream signaling changes, such as the arrestin pathway, intracellular Ca^2+^, and cAMPs that indirectly modulate ion channels. The original agonist is clozapine-N-oxide (CNO), a derived metabolite of clozapine, which is used as an antipsychotic. Thereafter, it was found that CNO can be converted into clozapine and cause psychoactive side effects in murine models [241]. Subsequently, more potent and specific agonists were invented, including JHU37152, JHU37160, and deschloroclozapine (DCZ) [242,243]. In addition to the choice of agonist, the diverse signaling pathways downstream of receptors make electricity manipulation less straightforward. Furthermore, the biological reactions could be variable in different cell types. Recent progress on DREADD structure activation is helpful for us to understand the mechanisms of their activation [244]. Still, careful experimental design was proposed to overcome the two potential issues in animal models [245]. Despite these weaknesses, the DREADD tools have found their way into various animal models, including flies, mice, rats, and primates [246]. In addition, DREADDs have also been extended to other research fields, including diabetes and endocrinology [247,248]. However, they have not been implemented in zebrafish yet. One study tried to utilize DREADD in zebrafish by microinjections, but failed in the endeavor [249]. Instead, they demonstrated that transient receptor potential (TRP) channels worked in the zebrafish embryos, and successfully manipulated Rohon–Beard and trigeminal sensory neurons using *islet-1* enhancer-driven TRP channels [249]. TRPV1 was activated by capsaicin, TRPM8 was activated by adding menthol, while TRPA1 activity required temperatures above 28 °C. Activation of TRPs induced dose-dependent locomotion and ablation, and altered wake-sleep behaviors [249]. In addition, the TRPV1-based approach was verified in another zebrafish study for modulating calcium flux in neutrophil [150]. Our laboratory has applied one of the DREADD, hM4DGi, to zebrafish embryos and larvae, and was able to change melanophore pigment cell dispersion [250]. Although the voltage reporter efficiency still needs to be improved, our results suggest that DREADD can be applied to zebrafish bioelectricity research.

PSAMs (pharmacologically selective actuator modules) are another set of chemogenetic tools. PSAMs share a similar key-and-lock concept, utilizing artificial inert molecules as agonists. For the actuator, instead of using mutated GPCRs as a lock, PSAMs use ligand-gated ion channels, such as nicotinic receptors (nAChR), serotonin receptor 3 (5HT3), GABA receptors, and the glycine receptor (GlyR). Each PSAM has a ligand binding domain (LBD) and a channel. These are mutated ligand-gated ion channels that can only be activated by PSEMs (pharmacologically selective effector molecules) [251,252]. Recently, a ligand-binding domain was genetically engineered, and more potent agonists were identified. Thus, this ultrapotent system is helpful for research and suitable for therapeutic applications, as exampled in mice and monkeys [251]. The first generation PSAM-GlyR was expressed in zebrafish horizontal cells (HCs), which connect rod and cone photoreceptors via synapses [253]. Disrupting Vm of HCs resulted in altered light response and lateral inhibition in retinal ganglion cells. This study illustrated that the ultrapotent PSAM-PSEM system could be extended for zebrafish bioelectric research.

In addition to the chemogenetic and optogenetic tools mentioned above, direct genetic modification, i.e., adding or deleting an ion channel regulator, is also achievable with the established tol2-transposon transgenic system and CRISPR technology [254]. This has already been demonstrated, such as in the transgenic Tg(*−5.4k-pax3a*: *kcnj13*-IRES-*EGFP*), where transient ectopic expression of *kcnj13* in zebrafish dermomyotome causes a long-finned phenotype in adults [112]. Nevertheless, this approach depends on already-known ion channel regulators and an available tissue-specific promoter. In addition, this genetic modification cannot be turned on and off, as with chemogenetic and optogenetic tools. Nevertheless, these tractable genetic tools are critical for implanting GEVIs, chemogenetics, and optogenetics into zebrafish.

**Table 4:** Common optogenetic and chemogenetic tools.

Optogenetic Tools					Chemogenetic Tools				
Name	Activation Method	Activation Result	Tested in Zebrafish	References	Name	Activation Method	Activation Result	Tested in Zebrafish	References
ChR2	Blue light (470 nm)	Depolarization	Melanophores, hair-cells, neurons	[141,230,235,255]	hM4DGi	DREADD agonists	Hyperpolarization	Melanophore	[222,250]
eNpHR3.0	Yellow light (590 nm)	Hyperpolarization	Neurons	[141,256]	hM3DGq	DREADD agonists	Depolarization		[222]
CoChR	Blue light (470 nm)	Depolarization	Neurons	[141,257]	hM3DGs	DREADD agonists	Depolarization		[222]
GtACR1	Green light (515 nm)	Hyperpolarization	Neurons, heart	[141,258,259,260]	KORD	DREADD agonists	Hyperpolarization		[222]
GtACR2	Blue light (470 nm)	Hyperpolarization	Neurons, heart	[141,259,260,261]	PSAM-5HT3-HC	PSEM ligands	Depolarization	Horizontal cells	[251]
BLINK2	Blue light (455 nm)	Hyperpolarization	Hair cells, lateral line neuromasts, neurons	[262]	PSAM-5HT3-LC	PSEM ligands	Depolarization	Horizontal cells	[251]
CheRiff	UV light (460 nm)	Depolarization	Neurons	[141,204]	PSAM-GlyR	PSEM ligands	Hyperpolarization	Horizontal cells	[251,253]
Chronos	Yellow light (500 nm)	Depolarization	Neurons	[141,263]	TRPV1	Capsaicin	Depolarization	Neurons, neutrophils	[150,249]
eArchT3.0	Yellow (570 nm)	Hyperpolarization	Neurons	[141,264]	TRPM8	Menthol	Depolarization	Neurons	[249]
ChrimsonR	Red light (590 nm)	Depolarization	Neurons	[141,265]	TRPA1	>28 °C	Depolarization	Neurons	[249]
					GluCl v2.0	Ivermectin	hyperpolarization		[266]

## 5. Prospects and Opportunities: Future Directions for Developmental Bioelectricity

The above-mentioned zebrafish fin and pigment genetic mutations are indirect evidence of bioelectricity in developmental patterning. Direct bioelectricity research in zebrafish is only possible now because of the recent availabilities of new voltage biosensors and manipulators. Although, here, we use zebrafish fin size and pigment cell patterns as examples, many other research directions can be pursued in this field. Below, we propose four major perspectives.

### 5.1. Systematic Zebrafish Embryo Bioelectricity Characterization

Though electrical signaling in neuronal tissues has been widely accepted and extensively investigated, other embryonic tissues remain completely unexplored. Characterizing non-neuronal tissues/cellular bioelectric signaling during embryogenesis is essentially the first step for deciphering the roles of bioelectricity. The in vivo real-time systematic characterization of vertebrate embryos has only just begun. Our Tg(*ubi:ASAP1*) fish line provided the first example of endogenous hyperpolarization signals of embryonic tissues [171]. More tissue-specific fish lines can be created with newly developed, more sensitive GEVIs, such as JEDI-2P, voltron2, Ace-mNeon2, and VARNAM2 using Tol2 transgenesis and CRISPR knock-in [178,182,215]. For example, in order to investigate somite cell contribution to the fins in our Dhi2059 mutant, somite compartments (dermatome, sclerotome, and syndetome) can be labeled with ASAP1 and other GEVIs to investigate the bioelectricity of these embryonic tissues in the future. On the other hand, faster and more sensitive imaging technologies, such as light-sheet fluorescence microscopes, have made this task more achievable [171,267,268]. It is possible that not only cellular Vm, but also its fluctuation amplitude, frequency, and rhythms, can serve as cell signals. Such tissue-specific, cell-type-specific, or subcellular bioelectric imaging in zebrafish embryos will reveal unprecedented insight into the functions of bioelectricity during embryonic development. Moreover, this information will be helpful in testing the theoretical concept of “the bioelectric code” [269,270,271]. In addition to GEVIs, biosensors for ions, such as the potassium sensor GINKO2 [272] and the chloride sensor ClopHensor [273], can also add another layer of information on bioelectricity.

### 5.2. Identifying Bioelectricity Contributing Genes and Redundancy of Ion Regulators

**Figure 3:**
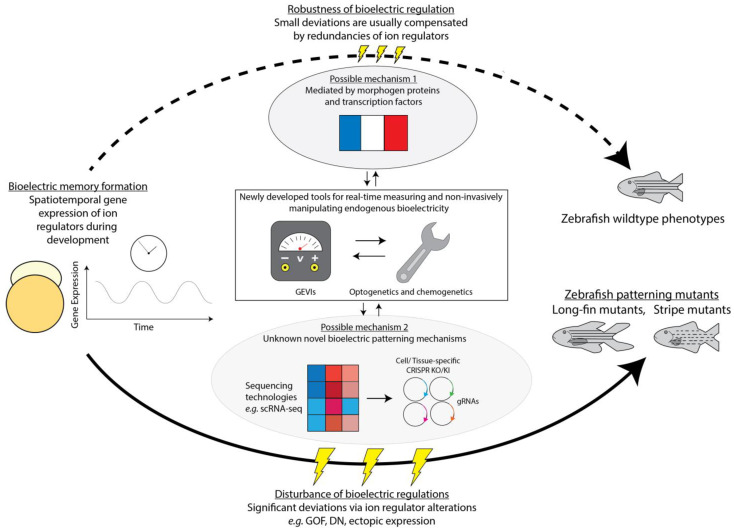
Summary and perspectives of bioelectricity in zebrafish developmental patterning research. Bioelectricity may function in zebrafish early embryos for patterning adult tissue/organs. Multiple ion regulators exist in each cell, so the cell’s bioelectric properties are generally robust. Minor disruptions (smaller lightning arrows) are not enough to change the bioelectric status quo, and only significant changes (big lightning arrows) may break through the robustness. This may explain why most zebrafish mutants were found with GOF (gain-of-function), DN (dominant negative), and ectopic expressions. Mechanistically, this bioelectric patterning may interact with already-known morphogen proteins and transcription factors. However, most likely, there are unknown mechanisms that mediate this bioelectric patterning. Next-generation sequencing technologies and CRISPR genome editing may help decipher such novel mechanisms. In addition, the recently developed genetically encoded tools for neuroscience, such as GEVIs, optogenetics, and chemogenetics, are readily adopted to the embryonic patterning research field. These tools allow us to monitor and manipulate bioelectricity in a non-invasive manner.

The cellular bioelectricity of a given cell type is composed of many ion regulators, as discussed above. However, major contributors of each cell type at a given embryonic stage also remain uncharacterized. Technically, the major challenge is ion regulator redundancy. Forward genetics starts with mutants created by random mutagenesis, and then addresses which gene is responsible for the mutant phenotype. This approach is powerful for identifying critical genes given a phenotype, especially embryonic development, in multiple model organisms, including flies, worms, mice, and zebrafish [94,274,275,276]. However, this approach might miss the underlying genes when genetic or biochemical redundancy exists. It is already known that multiple ion channels, connexins, pumps, and solute carriers can contribute to the overall cellular bioelectronic properties, though some are more prominent than others, given a cell type. There are 817 channel/transporter proteins known in the human genome in the cell membrane [277]. Many transporters underline many cellular functions, but also create redundancy that could make cellular bioelectric homeostasis very robust (Figure 3) [278]. Thus, it is unsurprising that only a limited number of zebrafish mutants were identified with altered fin and pigment patterns. Thus far, all the known fin-size mutations are genetically dominant. Most of them are either ectopically expressed (*kcnj13*, *kcnh2a*) or gain-of-function mutations (*kcnk5b, aqp3a,* and *lat4a*) (Table 2). This ismost likely because the loss of one ion regulator is generally insufficient to cause overall cellular bioelectric change. However, overexpression or gained function of ion regulators could drive the cell’s bioelectric property out of its physiological range (Figure 3). Thus, this type of mutant displays a developmental patterning phenotype. Dominant negative mutations of ion regulators may also function by interrupting structurally similar proteins in the same family. For example, the *kcc4a* in the *schleier* mutation could be interpreted as a dominant negative form, since the null mutant has no fin phenotype [108]. This redundancy not only leads to no phenotype, but also makes the experimental interpretation difficult. What is the solution to overcome the redundancy? With single-cell sequencing, it is feasible now to profile all the enriched ion regulators given a cell type [279,280]. For example, abundant ion regulators in different cell types within zebrafish somites and fins can be identified using this technique. Alternatively, whole mount in situ hybridization can be performed systematically, to examine their expression during zebrafish embryogenesis, as was performed in the case of the potassium channel gene subfamilies [281,282]. Once their gene expression is known, cell- and/or tissue-specific multiplexing gene knockout or knockdown by CRISPR can be used to examine certain ion regulators’ function in developmental patterning [283,284,285,286,287]. Moreover, chemogenetic and optogenetic tools are also expected to be effective to override endogenous ion regulators in a treated time window (Figure 3).

### 5.3. Developmental Patterning by Bioelectric Memory

Another fundamental question about bioelectricity is how it works during embryogenesis. From the current data of the zebrafish pigment mutants, cell–cell interactions are critical for adult pigment patterning. Moreover, the interaction between the pigment cells and other skin cells could also be important, as suggested by the transplant experiment of the *mau* fish [110]. One key phenomenon of bioelectric patterning we have learned from the Dhi2059 long-finned mutant is that the adult fin patterning can be determined much earlier, even back to the 1–2 dpf fish embryo stages [112]. The zebrafish larva fin buds have not yet developed at this embryonic stage. Very likely, the transient alteration of bioelectric properties of the two waves of fin progenitor cells, which migrate from somites into the fin buds [288,289], is enough to change adult zebrafish fin size. This memory of bioelectric property change may influence the differentiated cell interaction later in the fin anlagens. This bioelectric memory concept has been proposed and validated in the head–tail body axis determination in flatworms by the Levin research group [290]. Depolarizing edema cells during the first three hours after amputation is enough to cause a double-headed phenotype in flatworms, indicating that bioelectric memory is critical for patterning [291]. Thus, it is also reasonable that the bioelectric memory could be affected in other reported long-finned mutants [105,107,108]. Based on this, we have proposed a two-stage model for fin-size regulation [112]: the bioelectric memory may be formed at the somite stage, before the fin progenitor cells migrate to the fin buds. Furthermore, this memory can guide the local cell interactions and eventually determine the fin shape and size. However, the bioelectric memory can also make the developmental patterning mechanism more elusive. Generally, there will be a time lag for the phenotype development after bioelectric perturbation. Moreover, the bioelectric memory formation might occur within a short time window. Therefore, careful experimental design, including cell lineage tracking, bioelectricity measurement, and perturbation, is essential to decipher this intriguing mechanism.

### 5.4. Biological Pathways Downstream of Bioelectricity in Different Systems

Discovering unknown downstream signaling pathways of Vm on different functions could help explain all the diverse functions of bioelectricity. There is still a long way before we know how this biophysical cue is integrated into current biochemistry theory. As neural action potentials can deliver a signal to a long-range destination along the axon and relay by neurotransmitters, the non-excitatory embryonic tissues may use a similar mechanism in an atypical way for guiding cell–cell interactions. Instead, the embryonic cells may form weak bioelectrical networks [8]. Different cells and tissues may dominantly utilize specific pathways.

The morphogen protein gradient is one of the most influential developmental patterning principles with solid experimental support from many organisms, including zebrafish [292,293]. Relationships between bioelectricity and morphogen proteins, and their downstream transcription factors, are particularly interesting (Figure 3). This information will help us better understand how bioelectricity works and its interaction or cross-talks with current already-known morphogen proteins. No clear experimental evidence was reported in zebrafish yet, but multiple morphogen-signaling pathways were directly or indirectly linked with bioelectricity in other organisms. For example, cell membrane resting potential can alter a frog’s brain development through Notch signaling [294]. BMP signaling also mediates morphological changes of *Kcnj2/Kir2.1* gene mutations in both flies and mice [295,296,297]. However, the ion channel and WNT signaling relationship are less clear in developmental biology, and there is limited evidence from pathological conditions [298]. The most apparent case is bioelectricity and the hedgehog-signaling pathway. Two transient receptor potential (TRP) channels, PKD1L1 and PKD2L1, modulate ciliary calcium concentration, and the loss of these two channels leads to increased Gli1 activity and subsequent hedgehog signaling [299,300]. Additionally, direct evidence was reported that optogenetic depolarization was found to promote smoothened membrane localization and increase hedgehog signaling, which also promotes cellular depolarization in the fly wing disc [301]. Furthermore, two critical components of this pathway, DISP1 and PTCH1, function as cation-powered transporters. DISP1 utilizes the transmembrane sodium ion gradient to release cholesterylated SHH from HEK293 cells. In comparison, the PTCH1 receptor behaves as a K^+^-powered cholesterol transporter, which employs a transmembrane potassium ion gradient to antagonize SMO with cholesterol in NIH-3T3 cells [302]. Another report found the PTCH1 inhibits SMO and depends on extracellular sodium ion concentration [303]. Thus, bioelectricity may modulate multiple steps of hedgehog signaling from SHH secretion (establishment of morphogen gradients) to SHH reception (interpretation of morphogen), indicating bioelectricity patterning may be mediated by morphogen-signaling pathways, at least partially.

## 6. Conclusions

Bioelectricity has emerged as a new player in developmental patterning and organ size control. Here, we focused on the zebrafish model system and systematically reviewed genetic evidence of bioelectricity from zebrafish mutants. Additionally, we briefly summarized the newly developed genetically encoded voltage indicators and cellular voltage manipulators (optogenetics and chemogenetics) and their potential to be used for zebrafish bioelectricity research. Finally, we discussed future directions and opportunities for bioelectricity research in developmental patterning.

## Figures and Tables

**Table 1:** Reported cellular membrane potential of common vertebrate cell types.

Somatic Cells	Embryonic Origin	Millivolts (mV)	References
Skeletal myocyte	Paraxial mesoderm	From −91 to −65	[42]
Heart myocyte	Lateral plate mesoderm	From −95 to −40	[43]
Gut smooth muscle myocyte	Lateral plate mesoderm	From −70 to −35	[44]
Gliocyte	Neuroectoderm or neural crest	About −80	[45]
Neuron	Neuroectoderm	From −85 to −65	[46,47]
Adrenal cortex	Intermediate mesoderm	From −71 to −66	[48]
Adrenal medulla	Neural crest	From −32 to −20	[48]
Lymphocyte	Mesoderm	From −70 to −50	[49]
Thyroid follicular cell	Foregut endoderm	From −70 to −60	[50]
Chondrocyte	Mesoderm and neural crest	From −64 to −48	[51]
Fibroblast	All three embryological germ layers	From −25 to −16	[52,53]
Liver hepatocyte	Ventral foregut endoderm	From −50 to −20	[54]
Pancreas β-cell	Foregut endoderm	From −80 to −60	[35]
Epithelial cell	All three embryological germ layers	From −70 to −20	[55]
Melanocyte	Neural crest	From −50 to −40	[56,57,58]
White adipocyte	Mesoderm and neuroectoderm	From −69 to −17	[59]
Osteocytes	Mesoderm and neural crest	About −60	[60]
Cancer and tumor cells	All three embryological germ layers	From −50 to −5	[10,56,61,62]

## Data Availability

Not applicable.
